# Q-Neutrosophic Soft Relation and Its Application in Decision Making

**DOI:** 10.3390/e20030172

**Published:** 2018-03-06

**Authors:** Majdoleen Abu Qamar, Nasruddin Hassan

**Affiliations:** School of Mathematical Sciences, Faculty of Science and Technology, Universiti Kebangsaan Malaysia, Bangi 43600, Malaysia

**Keywords:** Q-neutrosophic soft set, Q-neutrosophic soft relation, neutrosophic soft set, fuzzy set

## Abstract

Q-neutrosophic soft sets are essentially neutrosophic soft sets characterized by three independent two-dimensional membership functions which stand for uncertainty, indeterminacy and falsity. Thus, it can be applied to two-dimensional imprecise, indeterminate and inconsistent data which appear in most real life problems. Relations are a suitable tool for describing correspondences between objects. In this study we introduce and discuss Q-neutrosophic soft relations, which can be discussed as a generalization of fuzzy soft relations, intuitionistic fuzzy soft relations, and neutrosophic soft relations. Q-neutrosophic soft relation is a sub Q-neutrosophic soft set of the Cartesian product of the Q-neutrosophic soft sets, in other words Q-neutrosophic soft relation is Q-neutrosophic soft sets in a Cartesian product of universes. We also present the notions of inverse, composition of Q-neutrosophic soft relations and functions along with some related theorems and properties. Reflexivity, symmetry, transitivity as well as equivalence relations and equivalence classes of Q-neutrosophic soft relations are also defined. Some properties of these concepts are presented and supported by real life examples. Finally, an algorithm to solve decision making problems using Q-neutrosophic soft relations is developed and verified by an example to show the efficiency of this method.

## 1. Introduction

The theory of neutrosophic set was firstly proposed by Smarandache [1] as a generalization of fuzzy set [2] and intuitionistic fuzzy set [3]. Neutrosophic set is a tri-component logic set, thus it can deal with uncertain, indeterminate and incompatible information where the indeterminacy is quantified explicitly and truth membership, indeterminacy membership and falsity membership are completely independent. The neutrosophic set was introduced for the first time by Smarandache in his 1998 book [4]. Neutrosophic set can handle indeterminate data which were not taken into account by fuzzy set theory, and intuitionistic fuzzy set theory.

Another commonly used method in handling uncertainties and representing incomplete and unreliable data is soft set theory which was established by Molodtsov [5] as a general mathematical tool used to handle uncertainties, imprecision and vagueness. Since its inception, a lot of extensions of soft set model have been developed such as fuzzy soft sets [6], vague soft sets [7], interval-valued vague soft sets [8,9,10], soft expert sets [11], soft multi set theory [12] and neutrosophic soft set [13,14,15,16]. At present, soft set has attracted wide attention and made many achievements [17,18,19].

Q-fuzzy soft sets was established by Adam and Hassan [20,21]. The theory behind the development of Q-fuzzy soft sets is that in many instances a second dimension must be added to the expression of the membership value of an element or object. This concept was extended to Q-intuitionistic fuzzy soft set by Broumi [22] by adding a two-dimensional non-membership function. However, these models cannot deal with indeterminate information which appears in two-dimensional universal sets. Thus, the concept of the Q-neutrosophic soft sets (Q-NSSs) is established to combine the key features of soft sets and Q-neutrosophic sets. The Q-neutrosophic soft sets (Q-NSSs) model is an improved model of neutrosophic sets that can represent two-dimensional information.

Since fuzzy set theory was introduced by Zadeh [2], it has been successfully applied to many real-life problems in uncertain and ambiguous environments [23,24,25], especially decision-making areas; fuzzy decision making has become a research focal point since then. Hence, the extensions of fuzzy set, intuitionistic fuzzy set [3], interval-valued [26], neutrosophic set [1], single valued neutrosophic soft set [27] and their hybrid models were widely applied to decision making problems. This includes neutrosophic sets and its extensions, to handle incomplete, indeterminate, and inconsistent problems in real life such as decision-making [28,29,30].

The utilization of fuzzy relations [2] derived from the observation that real life objects can be related to each other to a certain degree. Fuzzy relations are able to model vagueness, in the sense that they provide the degree to which two objects are related to each other. Nevertheless, they cannot model uncertainty. Consequently, Bustince and Burillo [31] introduced the concept of intuitionistic fuzzy relations followed by Dinda and Samanta [32] on intuitionistic fuzzy soft relations. This gives a way to include uncertainty to a certain degree, but it does not handle indeterminacy degree of membership. Hence, neutrosophic soft relations were initiated by Deli and Broumi [33]. Recently, many researchers studied fuzzy relations [34,35], fuzzy soft relations and their generalizations [36,37,38].

The relations between fuzzy sets, soft sets and their extensions have been widely studied. However, these relations do not encompass indeterminate information which appears in two dimensional universal sets. To overcome this, we introduce the Q-neutrosophic soft relation (Q-NSR), which represents the degree of presence, absence or indeterminacy of interaction between the elements of the Q-neutrosophic soft sets (Q-NSSs). Thus, it serves the indeterminacy and two-dimensionality of a data set at the same time, which cannot be served by fuzzy sets, soft sets and their extensions models. We present the concepts of inverse, functions and composition of Q-neutrosophic soft relations (Q-NSRs), some related theorems and properties. We define reflexivity, symmetry, transitivity as well as equivalence relations and equivalence classes of Q-neutrosophic soft relations (Q-NSRs). To show the ability of this model to solve decision making problems with two-dimensional indeterminate information, we developed an algorithm to solve decision making problems using Q-neutrosophic soft relations (Q-NSRs) and illustrate it by an example.

## 2. Preliminaries

In this section, we review the notions of soft sets, neutrosophic sets, neutrosophic soft sets with some of their properties which are pertinent to this work. The Q-neutrosophic soft sets (Q-NSSs) is also introduced.

Soft set theory was first introduced by Molodtsov [5] as a parametrized family of subsets of the universe of discourse *X*.

**Definition** **1** ([5]). *A pair (F,E) is called a soft set over X, if and only if F is a mapping of E into the set of all subsets of the set X. In other words, the soft set is a parametrized family of subsets of the set X.*

Neutrosophic set was established by Smarandache [1] as a generalization of fuzzy set [2], with a tri-component set to deal with uncertain, indeterminate and incompatible data.

**Definition** **2** ([1]). *A neutrosophic set *Γ* on the universe X is defined as*

$$\Gamma =\left\{\langle x,(T_{\Gamma }\left(x\right),I_{\Gamma }\left(x\right),F_{\Gamma }\left(x\right))\rangle :x\in X\right\},\;\mathrm{where}\;T,I,F:X\to {]}^{-}0,1^{+}[$$

*and*

$${}^{-}0\leq T_{\Gamma }\left(x\right)+I_{\Gamma }\left(x\right)+F_{\Gamma }\left(x\right)\leq 3^{+}.$$

**Definition** **3** ([39]). *Let *Γ* and *Ψ* be two neutrosophic sets, then we say that *Γ* is a subset of *Ψ* denoted by Γ⊆Ψ if and only if $T_{\Gamma }\left(x\right)\leq T_{\Psi }\left(x\right)$, $I_{\Gamma }\left(x\right)\geq I_{\Psi }\left(x\right)$ and $F_{\Gamma }\left(x\right)\geq F_{\Psi }\left(x\right)$ for all x∈X.*

Maji [13] presented the notion of neutrosophic soft sets as a generalization of soft sets. It is an improvement in the theory of soft sets and provides a way to deal with the uncertain data.

**Definition** **4** ([13]). *Let X be an initial universe set and E be a set of parameters. Consider A⊆E. Let P(X) denotes the set of all neutrosophic sets of X. The collection (F,A) is termed to be the soft neutrosophic set over X, where F is a mapping given by F:A→P(X).*

We will now introduce the concept of Q-neutrosophic set to provide a way to deal with uncertain, indeterminate and inconsistent two-dimensional information. We also extend this concept to multi Q-neutrosophic set and Q-neutrosophic soft set (Q-NSS).

**Definition** **5.** *Let X be a universal set and Q be a nonempty set. A Q-neutrosophic set $\Gamma_{Q}$ in X and Q is an object of the form*

$$\Gamma_{Q}=\left\{\langle \left(x,q\right),T_{\Gamma_{Q}}\left(x,q\right),I_{\Gamma_{Q}}\left(x,q\right),F_{\Gamma_{Q}}\left(x,q\right)\rangle :x\in X,q\in Q\right\},$$

*where $T_{\Gamma_{Q}},I_{\Gamma_{Q}},F_{\Gamma_{Q}}:X\times Q\to {]}^{-}0,1^{+}[$ are the true membership function, indeterminacy membership function and false membership function, respectively with ${}^{-}0\leq T_{\Gamma_{Q}}+I_{\Gamma_{Q}}+F_{\Gamma_{Q}}\leq 3^{+}$.*

Note that the set of all Q-neutrosophic sets over X will be denoted by QNS(X).

**Definition** **6.** *Let X be a universal set, Q be any nonempty set, l be any positive integer and I be a unit interval [0,1]. A multi Q-neutrosophic set ${\tilde{\Gamma }}_{Q}$ in X and Q is a set of ordered sequences*

$${\tilde{\Gamma }}_{Q}=\left\{\langle \left(x,q\right),T_{{\tilde{\Gamma }}_{Q{}_{i}}}\left(x,q\right),I_{{\tilde{\Gamma }}_{Q{}_{i}}}\left(x,q\right),F_{{\tilde{\Gamma }}_{Q{}_{i}}}\left(x,q\right)\rangle :x\in X,q\in Q\;\mathrm{for}\;\mathrm{all}\;i=1,2,\ldots ,l\right\},$$

*where $T_{{\tilde{\Gamma }}_{Q{}_{i}}},I_{{\tilde{\Gamma }}_{Q{}_{i}}},F_{{\tilde{\Gamma }}_{Q{}_{i}}}:X\times Q\to I^{l}\;\mathrm{for}\;\mathrm{all}\;i=1,2,\ldots ,l$ are respectively, truth membership function, indeterminacy membership function and falsity membership function for each x∈Xandq∈Q and satisfy the condition*

$$0\leq T_{{\tilde{\Gamma }}_{Q{}_{i}}}+I_{{\tilde{\Gamma }}_{Q{}_{i}}}+F_{{\tilde{\Gamma }}_{Q{}_{i}}}\leq 3\;\mathrm{for}\;\mathrm{all}\;i=1,2,\ldots ,l$$

*where l is called the dimension of ${\tilde{\Gamma }}_{Q}$.*

The set of all multi Q-neutrosophic sets of dimension l in X and Q is denoted by $\mu^{l}QNS\left(X\right)$.

**Definition** **7.** *Let X be a universal set, E be a set of parameters, and Q be a nonempty set. Let $\mu^{l}QNS\left(X\right)$ denote the set of all multi Q-neutrosophic sets on X with dimension l=1. Let A⊆E. A pair $\left(\Gamma_{Q},A\right)$ is called a Q-neutrosophic soft set (Q-NSS) over X, where $\Gamma_{Q}$ is a mapping given by*

$$\Gamma_{Q}:A\to \mu^{l}QNS\left(X\right)$$

*such that $\Gamma_{Q}\left(e\right)=\phi$ if e∉A. A Q-neutrosophic soft set (Q-NSS) can be represented by the set of ordered pairs*

$$\left(\Gamma_{Q},A\right)=\left\{\left(e,\Gamma_{Q}\left(e\right)\right):e\in A,\Gamma_{Q}\in \mu^{l}QNS\left(X\right)\right\}$$

The set of all Q-neutrosophic soft sets (Q-NSSs) in X and Q is denoted by QNSS(X).

**Definition** **8.** Let $\left(\Gamma_{Q},A\right),\left(\Psi_{Q},B\right)\in Q-NSS\left(X\right)$. Then $\left(\Psi_{Q},B\right)$ is a QNS subset of $\left(\Gamma_{Q},A\right)$, denoted by $\left(\Psi_{Q},B\right)\subseteq \left(\Gamma_{Q},A\right)$, if B⊆A and $\Psi_{Q}\left(x\right)\subseteq \Gamma_{Q}\left(x\right)$ for all x∈X.

The following example illustrates the above definition of the Q-NSS.

**Example** **1.** *Suppose we want to examine the attractiveness of houses that a person is considering purchasing. Suppose there are three houses in the universe $X=\{x_{1},x_{2},x_{3}\}$, Q={p,q} be a set of cities under consideration and $E=\{e_{1}=expensive,e_{2}=cheap\}$ be a set of decision parameters. Then the Q-NSS $\left(\Gamma_{Q},A\right)$ is given by:*

$$\begin{array}{cc} \left(\Gamma_{Q},A\right)=\{ & \langle e_{1},\left[\left(x_{1},p\right),0.7,0.3,0.4\right],\left[\left(x_{1},q\right),0.5,0.3,0.1\right],\left[\left(x_{2},p\right),0.9,0.7,0.6\right],\left[\left(x_{2},q\right),0.6,0.5,0.3\right]\rangle ,\\ & \langle e_{2},\left[\left(x_{1},p\right),0.3,0.4,0.8\right],\left[\left(x_{1},q\right),0.8,0.4,0.1\right],\left[\left(x_{2},p\right),0.5,0.5,0.8\right],\left[\left(x_{2},q\right),0.2,0.4,0.5\right]\rangle \}. \end{array}$$

The Q-NSS $\left(\Gamma_{Q},A\right)$ represents the influence of price on the degree of attraction of a house in a specific city. The neutrosophic components $T_{\Gamma_{Q_{i}}},I_{\Gamma_{Q_{i}}}$ and $F_{\Gamma_{Q_{i}}}$ represent the degree of true attractiveness, the degree of indeterminacy attractiveness and the the degree of falsity attractiveness of a house in a specific city, respectively. The three neutrosophic components lie in [0,1]. Values of $T_{\Gamma_{Q_{i}}}$ close to zero implies that the price has a very little influence on the degree of true attractiveness of a house in a specific city whereas values of $T_{\Gamma_{Q_{i}}}$ close to one implies that the price has a strong influence on the degree of true attractiveness of a house in a specific city. Similarly, for values of $I_{\Gamma_{Q_{i}}}$ and $F_{\Gamma_{Q_{i}}}$ components.

## 3. Q-Neutrosophic Soft Set Relations

In this section, after introducing the Cartesian product of two Q-NSSs, we will characterize the idea of Q-NSR, and present two fundamental operations of Q-NSRs, namely inverse and composition with some essential properties.

In the following we define the Cartesian product of two Q-NSSs followed by an illustrative example.

**Definition** **9.** *If X is an initial universal set, Q is a nonempty set, E is a set of parameters, A,B⊆E and $\left(\Gamma_{Q},A\right)$ and $\left(\Psi_{Q},B\right)$ are Q-NSSs over the universe X, then the Cartesian product of $\left(\Gamma_{Q},A\right)$ and $\left(\Psi_{Q},B\right)$, denoted by $\left(\Gamma_{Q},A\right)\times \left(\Psi_{Q},B\right)$, is a Q-NSS $\left(\Lambda_{Q},C\right)$, where C=A×B and $\left(\Lambda_{Q},C\right)$ is defined as:*

$$\begin{array}{cc} \left(\Lambda_{Q},C\right) & =\left(\Gamma_{Q},A\right)\times \left(\Psi_{Q},B\right)\\ & =\{\langle \left(a,b\right),T_{\Lambda_{Q}\left(a,b\right)}\left(x,q\right),I_{\Lambda_{Q}\left(a,b\right)}\left(x,q\right),F_{\Lambda_{Q}\left(a,b\right)}\left(x,q\right)\rangle :\left(a,b\right)\in A\times B,\left(x,q\right)\in X\times Q\}, \end{array}$$

*where $T_{\Lambda_{Q}\left(a,b\right)}\left(x,q\right),I_{\Lambda_{Q}\left(a,b\right)}\left(x,q\right),F_{\Lambda_{Q}\left(a,b\right)}\left(x,q\right)$ are the truth, indeterminacy and falsity membership functions of $\left(\Lambda_{Q},A\times B\right)$ such that $T_{\Lambda_{Q}\left(a,b\right)}\left(x,q\right),I_{\Lambda_{Q}\left(a,b\right)}\left(x,q\right),F_{\Lambda_{Q}\left(a,b\right)}\left(x,q\right):X\times Q\to \left[0,1\right]$ and for all (x,q)∈X×Q and (a,b)∈A×B we have:*

$$\begin{array}{cc} T_{\Lambda_{Q}\left(a,b\right)}\left(x,q\right) & =\min\{T_{\Gamma_{Q}\left(a\right)}\left(x,q\right),T_{\Psi_{Q}\left(b\right)}\left(x,q\right)\},\\ I_{\Lambda_{Q}\left(a,b\right)}\left(x,q\right) & =\max\{I_{\Gamma_{Q}\left(a\right)}\left(x,q\right),I_{\Psi_{Q}\left(b\right)}\left(x,q\right)\},\\ F_{\Lambda_{Q}\left(a,b\right)}\left(x,q\right) & =\max\{F_{\Gamma_{Q}\left(a\right)}\left(x,q\right),F_{\Psi_{Q}\left(b\right)}\left(x,q\right)\}. \end{array}$$

**Example** **2.** *Suppose we have a set of students X={a,b}, with their academic degree Q={p=Bachelor,q=Master}, their field of study A={M=Math,Ph=Physics} and their scholarly achievement B={Ex=Exellent,G=Good,P=Poor}. Suppose $\left(\Gamma_{Q},A\right)$ and $\left(\Psi_{Q},B\right)$ are two Q-NSSs over X defined as:*

$$\begin{array}{cc} \left(\Gamma_{Q},A\right)=\{ & \Gamma_{Q}\left(M\right)=\left\{\left[\left(a,p\right),0.2,0.5,0.7\right],\left[\left(a,q\right),0.1,0.6,0.9\right],\left[\left(b,p\right),0.3,0.5,0.8\right],\left[\left(b,q\right),0.1,0.4,0.7\right]\right\},\\ & \Gamma_{Q}\left(Ph\right)=\left\{\left[\left(a,p\right),0.4,0.9,0.9\right],\left[\left(a,q\right),0.4,0.3,0.2\right],\left[\left(b,p\right),0.1,0.4,0.5\right],\left[\left(b,q\right),0.5,0.4,0.3\right]\right\}\}. \end{array}$$

$$\begin{array}{cc} \left(\Psi_{Q},B\right)=\{ & \Psi_{Q}\left(Ex\right)=\left\{\left[\left(a,p\right),0.4,0.4,0.5\right],\left[\left(a,q\right),0.3,0.4,0.8\right],\left[\left(b,p\right),0.9,0.4,0.2\right],\left[\left(b,q\right),0.3,0.3,0.2\right]\right\},\\ & \Psi_{Q}\left(G\right)=\left\{\left[\left(a,p\right),0.5,0.2,0.6\right],\left[\left(a,q\right),0.3,0.2,0.6\right],\left[\left(b,p\right),0.7,0.4,0.2\right],\left[\left(b,q\right),0.6,0.3,0.1\right]\right\},\\ & \Psi_{Q}\left(P\right)=\left\{\left[\left(a,p\right),0.6,0.5,0.4\right],\left[\left(a,q\right),0.5,0.7,0.9\right],\left[\left(b,p\right),0.4,0.2,0.1\right],\left[\left(b,q\right),0.4,0.3,0.5\right]\right\}\}. \end{array}$$

*The Cartesian product of $\left(\Gamma_{Q},A\right)$ and $\left(\Psi_{Q},B\right)$ is*

$$\begin{array}{cc} \left(\Lambda_{Q},C\right)=\{ & \Gamma_{Q}\left(M\right)\times \Psi_{Q}\left(Ex\right),\Gamma_{Q}\left(M\right)\times \Psi_{Q}\left(G\right),\Gamma_{Q}\left(M\right)\times \Psi_{Q}\left(P\right),\\ & \Gamma_{Q}\left(Ph\right)\times \Psi_{Q}\left(Ex\right),\Gamma_{Q}\left(Ph\right)\times \Psi_{Q}\left(G\right),\Gamma_{Q}\left(Ph\right)\times \Psi_{Q}\left(P\right)\}. \end{array}$$

*where elements will look like*

$$\Gamma_{Q}\left(M\right)\times \Psi_{Q}\left(EX\right)=\left\{\left[\left(a,p\right),0.2,0.5,0.7\right],\left[\left(a,q\right),0.1,0.6,0.9\right],\left[\left(b,p\right),0.3,0.5,0.8\right],\left[\left(b,q\right),0.1,0.4,0.7\right]\right\}.$$

Now, we introduce the relation between two Q-NSSs, followed by the definitions of the domain and the range of a Q-NSR with some illustrative examples.

**Definition** **10.** *If X is an initial universal set, Q is a nonempty set, E is a set of parameters, A,B⊆E and $\left(\Gamma_{Q},A\right)$ and $\left(\Psi_{Q},B\right)$ are Q-NSSs over the universe X, then a Q-NSR from $\left(\Gamma_{Q},A\right)$ to $\left(\Psi_{Q},B\right)$ is a Q-NS subset of $\left(\Gamma_{Q},A\right)\times \left(\Psi_{Q},B\right)$, and is of the form $\left(R_{Q},C\right)$, where C⊆A×B and $R_{Q}\left(a,b\right)\subseteq \Gamma_{Q}\left(a\right)\times \Psi_{Q}\left(b\right),\forall \left(a,b\right)\in C$. Thus $\left(R_{Q},C\right)$ can be represented as:*

$$\left(R_{Q},C\right)=\left\{\langle \left(a,b\right),T_{R_{Q}\left(a,b\right)}\left(x,q\right),I_{R_{Q}\left(a,b\right)}\left(x,q\right),F_{R_{Q}\left(a,b\right)}\left(x,q\right)\rangle :\left(a,b\right)\in C\subseteq A\times B,\left(x,q\right)\in X\times Q\right\},$$

*where for all (x,q)∈X×Q and (a,b)∈C⊆A×B,*

$$\begin{array}{c} T_{R_{Q}\left(a,b\right)}\left(x,q\right)=\min\left\{T_{\Gamma_{Q}\left(a\right)}\left(x,q\right),T_{\Psi_{Q}\left(b\right)}\left(x,q\right)\right\},\\ I_{R_{Q}\left(a,b\right)}\left(x,q\right)=\max\left\{I_{\Gamma_{Q}\left(a\right)}\left(x,q\right),I_{\Psi_{Q}\left(b\right)}\left(x,q\right)\right\},\\ F_{R_{Q}\left(a,b\right)}\left(x,q\right)=\max\left\{F_{\Gamma_{Q}\left(a\right)}\left(x,q\right),F_{\Psi_{Q}\left(b\right)}\left(x,q\right)\right\}. \end{array}$$

If $\left(R_{Q},C\right)$ is a Q-NSR from $\left(\Gamma_{Q},A\right)$ to $\left(\Gamma_{Q},A\right)$, then it is called a Q-NSR on $\left(\Gamma_{Q},A\right)$ and it can be defined in the parameterized form as follows.

If $\left(\Gamma_{Q},A\right)=\left\{\Gamma_{Q}\left(a_{1}\right),\Gamma_{Q}\left(a_{2}\right),\ldots \right\}$, then $\Gamma_{Q}\left(a_{1}\right)R_{Q}\Gamma_{Q}\left(a_{2}\right)$ if and only if $\Gamma_{Q}\left(a_{1}\right)\times \Gamma_{Q}\left(a_{2}\right)\in \left(R_{Q},C\right)$.

**Definition** **11.** Let R be a Q-NSR from $\left(\Gamma_{Q},A\right)$ to $\left(\Psi_{Q},B\right)$. Then the domain of R (domR) is defined as the Q-NSS $\left(D,A_{1}\right)$, where $A_{1}=\left\{a\in A:R_{Q}\left(a,b\right)\in R_{Q},\;\mathrm{for}\;\mathrm{some}\;b\in B\right\}$ and $D\left(a_{1}\right)=\Gamma_{Q}\left(a_{1}\right)$, for all $a_{1}\in A_{1}$. The range of R (ranR) is defined as the Q-NSS $\left(RG,B_{1}\right)$, where $B_{1}=\left\{b\in B:R_{Q}\left(a,b\right)\in R_{Q},\;\mathrm{for}\;\mathrm{some}\;a\in A\right\}$ and $RG\left(b_{1}\right)=\Psi_{Q}\left(b_{1}\right),$ for all $b_{1}\in B_{1}$.

**Example** **3.** *Reconsider Example 2 with a relation R from $\left(\Gamma_{Q},A\right)$ to $\left(\Psi_{Q},B\right)$ as follows:*

$$R=\left\{\Gamma_{Q}\left(M\right)\times \Psi_{Q}\left(Ex\right),\Gamma_{Q}\left(Ph\right)\times \Psi_{Q}\left(G\right)\right\}\subset \left(\Gamma_{Q},A\right)\times \left(\Psi_{Q},B\right).$$

Then $domR=(D,A_{1})$ where $A_{1}=\left\{M,Ph\right\}$ and $D\left(a\right)=\Gamma_{Q}\left(a\right),$ for all $a\in A_{1}$, and $ranR=(RG,B_{1})$ where $B_{1}=\left\{Ex,G\right\}$ and $RG\left(b\right)=\Psi_{Q}\left(b\right),$ for all $b\in B_{1}$.

**Definition** **12.** The identity relation $I_{\left(\Gamma_{Q},A\right)}$ on a Q-NSS $\left(\Gamma_{Q},A\right)$ is defined as $\Gamma_{Q}\left(a\right)I_{\left(\Gamma_{Q},A\right)}\Gamma_{Q}\left(b\right)$ if and only if a=b.

**Example** **4.** In Example 2, the relation $I_{\left(\Gamma_{Q},A\right)}=\left\{\Gamma_{Q}\left(M\right)\times \Gamma_{Q}\left(M\right),\Gamma_{Q}\left(Ph\right)\times \Gamma_{Q}\left(Ph\right)\right\}$ is an identity relation.

Now, we introduce the operations of inverse and composition of two Q-NSRs followed by examples and relevant theorems.

**Definition** **13.** *If $\left(\Gamma_{Q},A\right)$ and $\left(\Psi_{Q},B\right)$ are Q-NSSs over a soft universe X and $\left(R_{Q},C\right)$ is a Q-NSR relation from $\left(\Gamma_{Q},A\right)$ and $\left(\Psi_{Q},B\right)$, then The inverse of a Q-NSR R, $\left(R_{Q}^{-1},C^{-1}\right)$ is a Q-NSR relation and is defined as:*

$$R_{Q}^{-1}\left(a,b\right)=R_{Q}\left(b,a\right),\forall \left(a,b\right)\in C\subseteq A\times B.$$

It is to be noted that the inverse of R is defined by reversing the order of every pair belonging to R.

**Example** **5.** Reconsider R as in Example 3, where we would then have $R^{-1}=\{\Psi_{Q}\left(Ex\right)\times \Gamma_{Q}\left(M\right)$, $\Psi_{Q}\left(G\right)\times \Gamma_{Q}\left(Ph\right)\}$.

**Theorem** **1.** *Suppose $\left(\Gamma_{Q},A\right)$ and $\left(\Psi_{Q},B\right)$ are Q-NSSs over a universe X, and $\left(R_{Q},C\right)$ and $\left(Z_{Q},C\right)$ are Q-NSRs from $\left(\Gamma_{Q},A\right)$ to $\left(\Psi_{Q},B\right)$. Then the following results hold:*

*1.*  ${\left({\left(R_{Q},C\right)}^{-1}\right)}^{-1}=\left(R_{Q},C\right)$.
*2.*  If $\left(R_{Q},C\right)\subseteq \left(Z_{Q},C\right)$ then ${\left(R_{Q},C\right)}^{-1}\subseteq {\left(Z_{Q},C\right)}^{-1}$.

**Proof.**  ∀(a,b)∈C⊆A×B, we have

1. ${\left({\left(R_{Q}\right)}^{-1}\right)}^{-1}\left(a,b\right)=R_{Q}^{-1}\left(b,a\right)$, thus ${\left({\left(R_{Q},C\right)}^{-1}\right)}^{-1}=\left(R_{Q},C\right)$.
2. If $R_{Q}\left(a,b\right)\subseteq Z_{Q}\left(a,b\right)$, then $R_{Q}^{-1}\left(b,a\right)\subseteq Z_{Q}^{-1}\left(b,a\right)$, and thus ${\left(R_{Q},C\right)}^{-1}\subseteq {\left(Z_{Q},C\right)}^{-1}$. ☐

Next, we will propose the definition of the composition of Q-NSRs along with an illustrative example, followed by related theorem.

**Definition** **14.** *If $\left(\Gamma_{Q},A\right),\left(\Psi_{Q},B\right)$ and $\left(\Upsilon_{Q},C\right)$ are Q-NSSs over a universe X and $\left(R_{Q},D_{1}\right)$ and $\left(Z_{Q},D_{2}\right)$ are Q-NSRs from $\left(\Gamma_{Q},A\right)$ to $\left(\Psi_{Q},B\right)$ and from $\left(\Psi_{Q},B\right)$ to $\left(\Upsilon_{Q},C\right)$ respectively, where $D_{1}\subseteq A\times B$ and $D_{2}\subseteq B\times C$, then the composition of the Q-NSRs $\left(R_{Q},D_{1}\right)$ and $\left(Z_{Q},D_{2}\right)$ denoted by $Z_{Q}\circ R_{Q}$ from $\left(\Gamma_{Q},A\right)$ to $\left(\Upsilon_{Q},C\right)$ is defined as:*

$$\begin{array}{cc} \left(Z_{Q}\circ R_{Q}\right)\left(a,c\right)=\{\langle & \left(a,c\right),T_{\left(Z_{Q}\circ R_{Q}\right)\left(a,c\right)}\left(x,q\right),I_{\left(Z_{Q}\circ R_{Q}\right)\left(a,c\right)}\left(x,q\right),F_{\left(Z_{Q}\circ R_{Q}\right)\left(a,c\right)}\left(x,q\right)\rangle :\\ & (a,c)\in A\times C,(x,q)\in X\times Q\}, \end{array}$$

*where for all $\left(a,b\right)\in D_{1}\subseteq A\times B$ and $\left(b,c\right)\in D_{2}\subseteq B\times C$,*

$$\begin{array}{cc} T_{{\left(Z_{Q}\circ R_{Q}\right)}_{Q}\left(a,c\right)}\left(x,q\right) & =\max\{T_{R_{Q}\left(a,b\right)}\left(x,q\right),T_{Z_{Q}\left(b,c\right)}\left(x,q\right)\}\\ & =\max\{\min\left\{T_{\Gamma_{Q}\left(a\right)}\left(x,q\right),T_{\Psi_{Q}\left(b\right)}\left(x,q\right)\right\},\min\left\{T_{\Psi_{Q}\left(b\right)}\left(x,q\right),T_{\Upsilon_{Q}\left(b\right)}\left(x,q\right)\right\}\},\\ I_{{\left(Z_{Q}\circ R_{Q}\right)}_{Q}\left(a,c\right)}\left(x,q\right) & =\min\{I_{R_{Q}\left(a,b\right)}\left(x,q\right),I_{Z_{Q}\left(b,c\right)}\left(x,q\right)\}\\ & =\min\{\max\left\{I_{\Gamma_{Q}\left(a\right)}\left(x,q\right),I_{\Psi_{Q}\left(b\right)}\left(x,q\right)\right\},\max\left\{I_{\Psi_{Q}\left(b\right)}\left(x,q\right),I_{\Upsilon_{Q}\left(b\right)}\left(x,q\right)\right\}\},\\ F_{{\left(Z_{Q}\circ R_{Q}\right)}_{Q}\left(a,c\right)}\left(x,q\right) & =\min\{F_{R_{Q}\left(a,b\right)}\left(x,q\right),F_{Z_{Q}\left(b,c\right)}\left(x,q\right)\}\\ & =\min\{\max\left\{F_{\Gamma_{Q}\left(a\right)}\left(x,q\right),F_{\Psi_{Q}\left(b\right)}\left(x,q\right)\right\},\max\left\{F_{\Psi_{Q}\left(b\right)}\left(x,q\right),F_{\Upsilon_{Q}\left(b\right)}\left(x,q\right)\right\}\}. \end{array}$$

**Example** **6.** Let X={s,t} be a set of students, Q={p,q} is the nationality of the students, E be a set of parameters and A,B,C⊆E, where $A=\{a_{1}=Harvard,a_{2}=Oxford,a_{3}=Cambridge\}$ describes the universities from which students may acquire degrees, $B=\{b_{1}=Master,b_{2}=PhD\}$ their academic degree and $C=\{c_{1}=Lecturer,c_{2}=Manager\}$ the professions students may be engaged in after acquiring degrees.

*Suppose that $\left(\Gamma_{Q},A\right),\left(\Psi_{Q},B\right)$ and $\left(\Upsilon_{Q},C\right)$ are Q-NSSs defined as:*

$$\begin{array}{cc} \left(\Gamma_{Q},A\right)=\{ & \Gamma_{Q}\left(a_{1}\right)=\left\{\left[\left(s,p\right),0.4,0.2,0.1\right],\left[\left(s,q\right),0.8,0.4,0.1\right],\left[\left(t,p\right),0.9,0.2,0.4\right],\left[\left(t,q\right),0.5,0.4,0.6\right]\right\},\\ & \Gamma_{Q}\left(a_{2}\right)=\left\{\left[\left(s,p\right),0.2,0.5,0.4\right],\left[\left(s,q\right),0.5,0.3,0.1\right],\left[\left(t,p\right),0.6,0.1,0.8\right],\left[\left(t,q\right),0.4,0.4,0.7\right]\right\},\\ & \Gamma_{Q}\left(a_{3}\right)=\left\{\left[\left(s,p\right),0.5,0.8,0.3\right],\left[\left(s,q\right),0.9,0.3,0.3\right],\left[\left(t,p\right),0.3,0.8,0.6\right],\left[\left(t,q\right),0.7,0.2,0.7\right]\right\}\}. \end{array}$$

$$\begin{array}{cc} \left(\Psi_{Q},B\right)=\{ & \Psi_{Q}\left(b_{1}\right)=\left\{\left[\left(s,p\right),0.4,0.3,0.2\right],\left[\left(s,q\right),0.3,0.2,0.3\right],\left[\left(t,p\right),0.5,0.6,0.3\right],\left[\left(t,q\right),0.7,0.8,0.2\right]\right\},\\ & \Psi_{Q}\left(b_{2}\right)=\left\{\left[\left(s,p\right),0.6,0.8,0.9\right],\left[\left(s,q\right),0.9,0.1,0.1\right],\left[\left(t,p\right),0.4,0.3,0.3\right],\left[\left(t,q\right),0.5,0.6,0.2\right]\right\}\}. \end{array}$$

$$\begin{array}{cc} \left(\Upsilon_{Q},C\right)=\{ & \Upsilon_{Q}\left(c_{1}\right)=\left\{\left[\left(s,p\right),0.7,0.1,0.6\right],\left[\left(s,q\right),0.5,0.1,0.4\right],\left[\left(t,p\right),0.4,0.3,0.4\right],\left[\left(t,q\right),0.9,0.2,0.4\right]\right\},\\ & \Upsilon_{Q}\left(c_{2}\right)=\left\{\left[\left(s,p\right),0.7,0.9,0.2\right],\left[\left(s,q\right),0.8,0.4,0.5\right],\left[\left(t,p\right),0.4,0.4,0.1\right],\left[\left(t,q\right),0.2,0.5,0.2\right]\right\}\}. \end{array}$$

*Define the Q-NSRs $R_{Q}$ from $\left(\Gamma_{Q},A\right)$ to $\left(\Psi_{Q},B\right)$ as a student from Oxford university or Cambridge university to investigate the effect of university on the master academic degree and $Z_{Q}$ from $\left(\Psi_{Q},B\right)$ to $\left(\Upsilon_{Q},C\right)$ as a master student to investigate the effect of the academic degree on the lecturing profession. Then the Q-NSRs $R_{Q}$ and $Z_{Q}$ are given by:*

$$\begin{array}{cc} R_{Q}=\{ & \langle (a_{2},b_{1}),[(s,p),0.2,0.5,0.4],[(s,q),0.3,0.3,0.3],[(t,p),0.5,0.6,0.8],[(t,q),0.4,0.8,0.7]\rangle ,\\ & \langle \left(a_{3},b_{1}\right),[(s,p),0.4,0.8,0.3],[(s,q),0.3,0.3,0.3],[(t,p),0.3,0.8,0.6],[(t,q),0.7,0.8,0.7]\rangle \}. \end{array}$$

$$Z_{Q}=\left\{\langle \left(b_{1},c_{1}\right),\left[\left(s,p\right),0.4,0.3,0.6\right],\left[\left(s,q\right),0.3,0.2,0.4\right],\left[\left(t,p\right),0.4,0.6,0.4\right],\left[\left(t,q\right),0.7,0.8,0.4\right]\rangle \right\}.$$

The relation $R_{Q}$ describes the effect of the university on being a master students, where it measures the true, indeterminacy and falsity degrees for a student to be a master’s student if he studied at Oxford university or Cambridge university. Whereas, the relation $Z_{Q}$ describes the effect of the master academic degree in engaging in a lecturing profession, it measures the true, indeterminacy and falsity degrees for a master’s student in engaging in a lecturing profession.

*The composition between the Q-NSRs $R_{Q}$ and $Z_{Q}$ which represents students engaged in a lecturing profession illustrates how to employ both components of parameters to convey the idea of the composition concept. The composition between the Q-NSRs $R_{Q}$ and $Z_{Q}$ is:*

$$\begin{array}{cc} Z_{Q}\circ R_{Q}=\{ & \langle \left(a_{2},c_{1}\right),\left[\left(s,p\right),0.4,0.3,0.4\right],\left[\left(s,q\right),0.3,0.2,0.3\right],\left[\left(t,p\right),0.5,0.6,0.4\right],\left[\left(t,q\right),0.7,0.8,0.4\right]\rangle ,\\ & \langle \left(a_{3},c_{1}\right),\left[\left(s,p\right),0.4,0.3,0.3\right],\left[\left(s,q\right),0.3,0.2,0.3\right],\left[\left(t,p\right),0.4,0.6,0.4\right],\left[\left(t,q\right),0.7,0.8,0.4\right]\rangle \}. \end{array}$$

The components $T_{Z_{Q}\circ R_{Q}}\left(a,c\right),I_{Z_{Q}\circ R_{Q}}\left(a,c\right)$ and $F_{Z_{Q}\circ R_{Q}}\left(a,c\right)$ represent respectively, the degrees of true, indeterminacy and falsity engaging in a lecturing profession for a master’s student who acquires his/her degree from Oxford university or Cambridge university. Thus, for the parameter $\langle \left(a_{2},c_{1}\right)\rangle$ the term [(t,p),0.5,0.6,0.4] implies that student “t” whose nationality is “p” and studying for his/her master’s degree at Oxford university has a 0.5 truth degree of engaging in lecturing profession, 0.6 indeterminacy degree of engaging in a lecturing profession and 0.4 falsity degree of engaging in a lecturing profession.

**Theorem** **2.** If $\left(\Gamma_{Q},A\right),\left(\Psi_{Q},B\right)$ and $\left(\Upsilon_{Q},C\right)$ are Q-NSSs over X and $\left(R_{Q},D_{1}\right)$ and $\left(Z_{Q},D_{2}\right)$ are Q-NSRs from $\left(\Gamma_{Q},A\right)$ to $\left(\Psi_{Q},B\right)$ and $\left(\Psi_{Q},B\right)$ to $\left(\Upsilon_{Q},C\right)$ respectively, where $D_{1}\subseteq A\times B$ and $D_{2}\subseteq B\times C$, then ${\left(Z_{Q}\circ R_{Q}\right)}^{-1}=R_{Q}^{-1}\circ Z_{Q}^{-1}$.

**Proof.**  If $\left(R_{Q},D_{1}\right)$ and $\left(Z_{Q},D_{2}\right)$ are Q-NSRs from $\left(\Gamma_{Q},A\right)$ to $\left(\Psi_{Q},B\right)$ and $\left(\Psi_{Q},B\right)$ to $\left(\Upsilon_{Q},C\right)$ respectively, then $Z_{Q}\circ R_{Q}\subseteq \left(\Gamma_{Q},A\right)\times \left(\Upsilon_{Q},C\right)$. Now, for (a,c)∈A×C,(x,q)∈X×Q,

$$\begin{array}{cc} T_{{\left(Z_{Q}\circ R_{Q}\right)}^{-1}\left(c,a\right)}\left(x,q\right) & =T_{\left(Z_{Q}\circ R_{Q}\right)\left(a,c\right)}\left(x,q\right)\\ & =\max\{T_{R_{Q}\left(a,b\right)}\left(x,q\right),T_{Z_{Q}\left(b,c\right)}\left(x,q\right)\}\\ & =\max\{T_{Z_{Q}\left(b,c\right)}\left(x,q\right),T_{R_{Q}\left(a,b\right)}\left(x,q\right)\}\\ & =\max\{\min\left\{T_{\Psi_{Q}\left(b\right)}\left(x,q\right),T_{\Upsilon_{Q}\left(c\right)}\left(x,q\right)\right\},\min\left\{T_{\Gamma_{Q}\left(a\right)}\left(x,q\right),T_{\Psi_{Q}\left(b\right)}\left(x,q\right)\right\}\}\\ & =\max\{\min\left\{T_{\Upsilon_{Q}\left(c\right)}\left(x,q\right),T_{\Psi_{Q}\left(b\right)}\left(x,q\right)\right\},\min\left\{T_{\Psi_{Q}\left(b\right)}\left(x,q\right),T_{\Gamma_{Q}\left(a\right)}\left(x,q\right)\right\}\}\\ & =\max\left\{T_{Z_{Q}^{-1}\left(c,b\right)}\left(x,q\right),T_{R_{Q}^{-1}\left(b,a\right)}\left(x,q\right)\right\}=T_{R_{Q}^{-1}\circ Z_{Q}^{-1}\left(c,a\right)}\left(x,q\right). \end{array}$$

Similar results follow for the rest of the terms. This completes the proof. ☐

## 4. Partitions on Q-Neutrosophic Soft Sets

In this section, we will introduce various types of Q-NSRs, partitions and equivalence classes of Q-NSSs with some related theorems.

**Definition** **15.** *Let R be a relation on $\left(\Gamma_{Q},A\right)$, then*

*1.*  $\left(R_{Q},C\right)$ is reflexive if $R_{Q}\left(a,a\right)\in \left(R_{Q},C\right),\forall a\in A$.
*2.*  $\left(R_{Q},C\right)$ is symmetric if $R_{Q}\left(a,b\right)\in \left(R_{Q},C\right)\Rightarrow R_{Q}\left(b,a\right)\in \left(R_{Q},C\right),\forall \left(a,b\right)\in A\times A$.
*3*  $\left(R_{Q},C\right)$ is transitive if $R_{Q}\left(a,b\right)\in \left(R_{Q},C\right)\;\mathrm{and}\;R_{Q}\left(b,c\right)\in \left(R_{Q},C\right)\Rightarrow R_{Q}\left(a,c\right)\in \left(R_{Q},C\right)$, ∀a,b,c∈A.
*4*  $\left(R_{Q},C\right)$ is a Q-NS equivalence relation if it is reflexive, symmetric and transitive.

**Example** **7.** *Consider a Q-NSS $\left(\Gamma_{Q},A\right)$ over X, where $X=\left\{a,b\right\},Q=\left\{p,q\right\},A=\left\{e_{1},e_{2}\right\}$ and*

$$\begin{array}{cc} \Gamma_{Q}\left(e_{1}\right) & =\{[(a,p),0.2,0.5,0.7],[(b,p),0.1,0.2,0.9],[(a,q),0.3,0.5,0.2],[(a,q),0.1,0.4,0.8]\}\\ \Gamma_{Q}\left(e_{2}\right) & =\{[(a,p),0.3,0.5,0.6],[(a,q),0.4,0.5,0.8],[(b,p),0.7,0.1,0.5],[(b,q),0.5,0.5,0.6]\}. \end{array}$$

Consider a relation $\left(R_{Q},C\right)$ defined on $\left(\Gamma_{Q},A\right)$ as $\{\Gamma_{Q}\left(e_{1}\right)\times \Gamma_{Q}\left(e_{1}\right),\Gamma_{Q}\left(e_{1}\right)\times \Gamma_{Q}\left(e_{2}\right)$, $\Gamma_{Q}\left(e_{2}\right)\times \Gamma_{Q}\left(e_{1}\right)$, $\Gamma_{Q}\left(e_{2}\right)\times \Gamma_{Q}\left(e_{2}\right)\}$. This relation is a Q-NS equivalence relation.

**Definition** **16.** *Let $\left(\Gamma_{Q},A\right)$ be a Q-NSS. Then the equivalence class of $\Gamma_{Q}\left(a\right)$ is defined as*

$$\left[\Gamma_{Q}\left(a\right)\right]=\left\{\Gamma_{Q}\left(b\right):\Gamma_{Q}\left(b\right)R\Gamma_{Q}\left(a\right),\forall a,b\in A\right\}.$$

**Example** **8.** Reconsider R as in Example 3, we would then have $\left[\Gamma_{Q}\left(e_{1}\right)\right]=\left\{\Gamma_{Q}\left(e_{1}\right),\Gamma_{Q}\left(e_{2}\right)\right\}=\left[\Gamma_{Q}\left(e_{2}\right)\right]$.

**Lemma** **1.** Let $\left(R_{Q},C\right)$ be an equivalence relation on a Q-NSS $\left(\Gamma_{Q},A\right)$. For any $\Gamma_{Q}\left(a\right),\Gamma_{Q}\left(b\right)\in \left(\Gamma_{Q},A\right)$, $\Gamma_{Q}\left(a\right)R_{Q}\Gamma_{Q}\left(b\right)$ if and only if $\left[\Gamma_{Q}\left(a\right)\right]=\left[\Gamma_{Q}\left(b\right)\right]$.

**Proof.**  Suppose $\left[\Gamma_{Q}\left(a\right)\right]=\left[\Gamma_{Q}\left(b\right)\right]$. Since $\left(R_{Q},C\right)$ is reflexive, then $\Gamma_{Q}\left(b\right)R_{Q}\Gamma_{Q}\left(b\right)$, hence $\Gamma_{Q}\left(b\right)\in \left[\Gamma_{Q}\left(b\right)\right]=\left[\Gamma_{Q}\left(a\right)\right]$ which gives $\Gamma_{Q}\left(a\right)R_{Q}\Gamma_{Q}\left(b\right)$.

Conversely, suppose $\Gamma_{Q}\left(a\right)R_{Q}\Gamma_{Q}\left(b\right)$. Let $\Gamma_{Q}\left(a_{1}\right)\in \left[\Gamma_{Q}\left(a\right)\right]$. Then $\Gamma_{Q}\left(a_{1}\right)R_{Q}\Gamma_{Q}\left(a\right)$. Using the transitive property of $\left(R_{Q},C\right)$ this gives $\Gamma_{Q}\left(a_{1}\right)\in \left[\Gamma_{Q}\left(b\right)\right]$. Hence, $\left[\Gamma_{Q}\left(a\right)\right]\subseteq \left[\Gamma_{Q}\left(b\right)\right]$. Similarly, $\left[\Gamma_{Q}\left(b\right)\right]\subseteq \left[\Gamma_{Q}\left(a\right)\right]$. Thus, $\left[\Gamma_{Q}\left(b\right)\right]=\left[\Gamma_{Q}\left(a\right)\right]$. ☐

Now, we define the partition of a Q-NSS followed by some related theorems.

**Definition** **17.** *A collection of nonempty Q-NS subsets $P=\{\left(\Gamma_{Q_{i}},A_{i}\right):i\in I\}$ of a Q-NSS $\left(\Gamma_{Q},A\right)$ is called a partition of $\left(\Gamma_{Q},A\right)$ such that*

*1.*  $\left(\Gamma_{Q},A\right)={\tilde{\cup }}_{i}\left(\Gamma_{Q_{i}},A_{i}\right)$ and
*2.*  $A_{i}\cap A_{j}=\phi$, whenever i≠j.

**Example** **9.** *Let X={a,b} be a universal set, Q={p,q} be a nonempty set, $A=\{e_{1},e_{2},e_{3}\}$ be a set of parameters and $\left(\Gamma_{Q},A\right)$ is a Q-NSS over X defined as:*

$$\begin{array}{cc} \left(\Gamma_{Q},A\right)=\{ & \Gamma_{Q}\left(e_{1}\right)=\left\{\left[\left(a,p\right),0.2,0.3,0.8\right],\left[\left(b,p\right),0.5,0.7,0.2\right],\left[\left(b,q\right),0.5,0.3,0.6\right]\right\},\\ & \Gamma_{Q}\left(e_{2}\right)=\left\{\left[\left(a,p\right),0.4,0.2,0.6\right],\left[\left(a,q\right),0.4,0.3,0.9\right]\right\},\\ & \Gamma_{Q}\left(e_{3}\right)=\left\{\left[\left(a,p\right),0.4,0.8,0.4\right],\left[\left(a,q\right),0.7,0.8,0.9\right],\left[\left(b,p\right),0.1,0.7,0.5\right],\left[\left(b,q\right),0.2,0.1,0.9\right]\right\}\}. \end{array}$$

Suppose $A_{1}=\left\{e_{1},e_{3}\right\}$, $A_{2}=\left\{e_{2}\right\},$ where $\left(\Gamma_{Q_{1}},A_{1}\right)=\left\{\Gamma_{Q_{1}}\left(e_{1}\right),\Gamma_{Q_{1}}\left(e_{3}\right)\right\}$ and $\left(\Gamma_{Q_{2}},A_{2}\right)=\left\{\Gamma_{Q_{2}}\left(e_{2}\right)\right\}$ are Q-Ns subsets of $\left(\Gamma_{Q},A\right)$ such that $\Gamma_{Q_{i}}=\Gamma_{Q},$ for i=1,2. Clearly, $A_{1}\cap A_{2}=\phi$ and $\left(\Gamma_{Q_{1}},A_{1}\right)\cup \left(\Gamma_{Q_{2}},A_{2}\right)=\left(\Gamma_{Q},A\right)$. Thus, $\left\{\left(\Gamma_{Q_{1}},A_{1}\right),\left(\Gamma_{Q_{2}},A_{2}\right)\right\}$ is a Q-neutrosophic soft set partition of $\left(\Gamma_{Q},A\right)$.

**Remark** **1.** Elements of the partition are called a block of $\left(\Gamma_{Q},A\right)$.

Corresponding to a partition $\left(\Gamma_{Q_{i}},A_{i}\right)$ of a Q-NSS $\left(\Gamma_{Q},A\right)$, we can define a Q-NSR on $\left(\Gamma_{Q},A\right)$ by $\Gamma_{Q}\left(a\right)R\Gamma_{Q}\left(b\right)$ iff $\Gamma_{Q}\left(a\right)$ and $\Gamma_{Q}\left(b\right)$ belong to the same block. Now, we will prove that the relation defined in this manner is an equivalence relation.

**Theorem** **3.** Let $\left\{\left(\Gamma_{Q_{i}},A_{i}\right),i\in I\right\}$ be a partition of the Q-NSS $\left(\Gamma_{Q},A\right)$. The Q-NSR defined on $\left(\Gamma_{Q},A\right)$ as $\Gamma_{Q}\left(a\right)R_{Q}\Gamma_{Q}\left(b\right)$ is an equivalence relation if and only if $\Gamma_{Q}\left(a\right)$ and $\Gamma_{Q}\left(b\right)$ are members of the same block.

**Proof.**  Reflexive: Let $\Gamma_{Q}\left(a\right)$ be any element of $\left(\Gamma_{Q},A\right)$. It is clear that $\Gamma_{Q}\left(a\right)$ is in the same block itself. Hence, $R_{Q}\left(a,a\right)\in \left(R_{Q},C\right)$.

Symmetric: If $R_{Q}\left(a,b\right)\in \left(R_{Q},C\right)$, then $\Gamma_{Q}\left(a\right)$ and $\Gamma_{Q}\left(b\right)$ are in the same block. Therefore, $R_{Q}\left(b,a\right)\in \left(R_{Q},C\right)$.

Transitive: If $R_{Q}\left(a,b\right)\in \left(R_{Q},C\right)$ and $R_{Q}\left(b,c\right)\in \left(R_{Q},C\right)$ then $\Gamma_{Q}\left(a\right)$, $\Gamma_{Q}\left(b\right)$ and $\Gamma_{Q}\left(c\right)$ must lie in the same block. Therefore, $R_{Q}\left(a,c\right)\in \left(R_{Q},C\right)$. ☐

**Remark** **2.** *The equivalence Q-NSR defined in the above theorem is called an equivalence relation determined by the partition P. In the previous example the equivalence relation determined by the partition $P=\{\left(\Gamma_{Q},A_{1}\right)$, $\left(\Gamma_{Q},A_{2})\right\}$ is given by*

$$R=\{\Gamma_{Q}\left(a_{1}\right)\times \Gamma_{Q}\left(a_{1}\right),\Gamma_{Q}\left(a_{2}\right)\times \Gamma_{Q}\left(a_{2}\right),\Gamma_{Q}\left(a_{3}\right)\times \Gamma_{Q}\left(a_{3}\right),\Gamma_{Q}\left(a_{1}\right)\times \Gamma_{Q}\left(a_{3}\right),\Gamma_{Q}\left(a_{3}\right)\times \Gamma_{Q}\left(a_{1}\right)\}.$$

**Theorem** **4.** Corresponding to every equivalence relation defined on a Q-NSS $\left(\Gamma_{Q},A\right)$ there exists a partition on $\left(\Gamma_{Q},A\right)$ and this partition precisely consists of the equivalence classes of $\left(R_{Q},C\right)$.

**Proof.**  Let $\left[\Gamma_{Q}\left(a\right)\right]$ be equivalence class with respect to a relation $\left(R_{Q},C\right)$ on $\left(\Gamma_{Q},A\right)$. Let $A_{a}$ be all elements in *A* corresponding to $\left[\Gamma_{Q}\left(a\right)\right]$ i.e., $A_{a}=\left\{b\in A:R_{Q}\left(b,a\right)\in \left(R_{Q},C\right)\right\}$. Thus we can denote $\left[\Gamma_{Q}\left(a\right)\right]$ as $\left(\Gamma_{Q},A_{a}\right)$. Thus, we have to show that the collection $\left\{\left(\Gamma_{Q},A_{a}\right):a\in A\right\}$ of such distinct sets forms a partition *P* of $\left(\Gamma_{Q},A\right)$. In order to do this we should prove

(i) $\left(\Gamma_{Q},A\right)={\tilde{\cup }}_{a\in A}\left(\Gamma_{Q},A_{a}\right),$
(ii) If $A_{a},A_{b}$ are not identical then $A_{a}\cap A_{b}=\phi$.

Since $R_{Q}$ is reflexive, $R_{Q}\left(a,a\right)\in \left(R_{Q},C\right)\forall a\in A$ so that $\left(\Gamma_{Q},A\right)={\tilde{\cup }}_{a\in A}\left(\Gamma_{Q},A_{a}\right)$.

Now for the second part, let $x\in A_{a}\cap A_{b}$. Then $\Gamma_{Q}\left(x\right)\in \left(\Gamma_{Q},A_{a}\right)$ and $\Gamma_{Q}\left(x\right)\in \left(\Gamma_{Q},A_{b}\right)$. This implies $R_{Q}\left(x,a\right)\in \left(R_{Q},C\right)$ and $R_{Q}\left(x,b\right)\in \left(R_{Q},C\right)$. Using the transitive property of $R_{Q}$, we have $R_{Q}\left(a,b\right)\in \left(R_{Q},C\right)$. Now, using lemma 1 we have $\left[\Gamma_{Q}\left(a\right)\right]=\left[\Gamma_{Q}\left(b\right)\right]$. This gives $A_{a}=A_{b}$ (contradiction) since $A_{a}$ and $A_{b}$ are not identical, hence $A_{a}\cap A_{b}=\phi$. ☐

**Remark** **3.** The partition constructed in the above theorem consisting of all equivalence classes of $\left(R_{Q},C\right)$ is called the quotient Q-NSS of $\left(\Gamma_{Q},A\right)$ and is denoted by $\left(\Gamma_{Q},A\right)/\left(R_{Q},C\right)$.

## 5. Q-Neutrosophic Soft Functions

In this section, we present the concept of Q-NS function and some special types of Q-neutrosophic soft functions with related theorems.

**Definition** **18.** Let $\left(\Gamma_{Q},A\right)$ and $\left(\Psi_{Q},B\right)$ be two nonempty Q-NSS. Then a Q-NSR f from $\left(\Gamma_{Q},A\right)$ to $\left(\Psi_{Q},B\right)$ is called a Q-NS function if every element in the domain has a unique element in the range. We write $f:\left(\Gamma_{Q},A\right)\to \left(\Psi_{Q},B\right)$. If $\Gamma_{Q}\left(a\right)f\Psi_{Q}\left(b\right)$ then $f\left(\Gamma_{Q}\left(a\right)\right)=\Psi_{Q}\left(b\right)$.

**Example** **10.** *Reconsider Example 2. The Q-NSR f which consists of a science student with excellent GPA forms a Q-NS function from $\left(\Gamma_{Q},A\right)$ to $\left(\Psi_{Q},B\right)$ as follows:* $f=\{\Gamma_{Q}\left(M\right)\times \Psi_{Q}\left(Ex\right),\Gamma_{Q}\left(Ph\right)\times \Psi_{Q}\left(Ex\right)\}$.

**Definition** **19.** *Let $f:\left(\Gamma_{Q},A\right)\to \left(\Psi_{Q},B\right)$ be a Q-NS function. Then*

*1.*  f is injective (one to one) if $\Gamma_{Q}\left(a_{1}\right)\neq \Gamma_{Q}\left(a_{2}\right)$ implying $f\left(\Gamma_{Q}\left(a_{1}\right)\right)\neq f\left(\Gamma_{Q}\left(a_{2}\right)\right)$ for $\Gamma_{Q}\left(a_{1}\right),\Gamma_{Q}\left(a_{2}\right)\in \left(\Gamma_{Q},A\right)$. i.e., f is injective if each element of ranf appears exactly once in the function.
*2.*  f is surjective (onto) if $f\left(\left(\Gamma_{Q},A\right)\right)=\left(\Psi_{Q},B\right)$ i.e., $ranf=(\Psi_{Q},B)$.
*3.*  f is bijective if it is both injective and surjective.
*4.*  f is a constant function if all elements in domf have the same image.
*5.*  f is an identity function if the identity Q-NS function I on a QNSS $\left(\Gamma_{Q},A\right)$ is defined by the function $I:\left(\Gamma_{Q},A\right)\to \left(\Gamma_{Q},A\right)$ as $I\left(\Gamma_{Q}\left(a\right)\right)=\Gamma_{Q}\left(a\right)$ for every $\Gamma_{Q}\left(a\right)$ in $\left(\Gamma_{Q},A\right)$.

**Theorem** **5.** *Let $f:\left(\Gamma_{Q},A\right)\to \left(\Psi_{Q},B\right)$ be a QNS function, $\left(\Gamma_{Q},A_{1}\right)$ and $\left(\Gamma_{Q},A_{2}\right)$ be a Q-NS subsets of $\left(\Gamma_{Q},A\right)$. Then*

*1.*  $\left(\Gamma_{Q},A_{1}\right)\subseteq \left(\Gamma_{Q},A_{2}\right)\Rightarrow f\left(\Gamma_{Q},A_{1}\right)\subseteq f\left(\Gamma_{Q},A_{2}\right)$.
*2.*  $f\left[\left(\Gamma_{Q},A_{1}\right)\cup \left(\Gamma_{Q},A_{2}\right)\right]=f\left(\Gamma_{Q},A_{1}\right)\cup f\left(\Gamma_{Q},A_{2}\right)$.
*3.*  $f\left[\left(\Gamma_{Q},A_{1}\right)\cap \left(\Gamma_{Q},A_{2}\right)\right]\subseteq f\left(\Gamma_{Q},A_{1}\right)\cap f\left(\Gamma_{Q},A_{2}\right)$ equality holds if f is one to one.

**Proof.**  1. Let $\Psi_{Q}\left(b\right)\in f\left(\Gamma_{Q},A_{1}\right)$. Then there exists $\Gamma_{Q}\left(a\right)\in \left(\Gamma_{Q},A_{1}\right)$ such that $f\left(\Gamma_{Q}\left(a\right)\right)=\Psi_{Q}\left(b\right)$. Since $\left(\Gamma_{Q},A_{1}\right)\subseteq \left(\Gamma_{Q},A_{2}\right)$, then $\Psi_{Q}\left(b\right)\in f\left(\Gamma_{Q},A_{2}\right)$. Therefore, $f\left(\Gamma_{Q},A_{1}\right)\subseteq f\left(\Gamma_{Q},A_{2}\right)$.

2. Let $\Psi_{Q}\left(b\right)\in f\left[\left(\Gamma_{Q},A_{1}\right)\cup \left(\Gamma_{Q},A_{2}\right)\right]$. Then $f\left(\Gamma_{Q}\left(a\right)\right)=\Psi_{Q}\left(b\right)$ such that $\Gamma_{Q}\left(a\right)\in \left[\left(\Gamma_{Q},A_{1}\right)\cup \left(\Gamma_{Q},A_{2}\right)\right]$. By using union definition, we have $\Gamma_{Q}\left(a\right)\in \left(\Gamma_{Q},A_{1}\right)$ or $\Gamma_{Q}\left(a\right)\in \left(\Gamma_{Q},A_{2}\right)$.

This implies, $\Psi_{Q}\left(b\right)\in f\left(\Gamma_{Q},A_{1}\right)$ or $\Psi_{Q}\left(b\right)\in f\left(\Gamma_{Q},A_{2}\right)$. Thus, $\Psi_{Q}\left(b\right)\in f\left(\Gamma_{Q},A_{1}\right)\cup f\left(\Gamma_{Q},A_{2}\right)$. Therefore, $f\left[\left(\Gamma_{Q},A_{1}\right)\cup \left(\Gamma_{Q},A_{2}\right)\right]\subseteq f\left(\Gamma_{Q},A_{1}\right)\cup f\left(\Gamma_{Q},A_{2}\right)$.

Now, clearly $\left(\Gamma_{Q},A_{1}\right)\subseteq \left(\Gamma_{Q},A_{1}\right)\cup \left(\Gamma_{Q},A_{2}\right)$ and $\left(\Gamma_{Q},A_{2}\right)\subseteq \left(\Gamma_{Q},A_{1}\right)\cup \left(\Gamma_{Q},A_{2}\right)$. This implies $f\left(\Gamma_{Q},A_{1}\right)\subseteq f\left[\left(\Gamma_{Q},A_{1}\right)\cup \left(\Gamma_{Q},A_{2}\right)\right]$ and $f\left(\Gamma_{Q},A_{2}\right)\subseteq f\left[\left(\Gamma_{Q},A_{1}\right)\cup \left(\Gamma_{Q},A_{2}\right)\right]$. Thus, $f\left(\Gamma_{Q},A_{1}\right)\cup f\left(\Gamma_{Q},A_{2}\right)\subseteq f\left[\left(\Gamma_{Q},A_{1}\right)\cup \left(\Gamma_{Q},A_{2}\right)\right]$. Hence, $f\left[\left(\Gamma_{Q},A_{1}\right)\cup \left(\Gamma_{Q},A_{2}\right)\right]=f\left(\Gamma_{Q},A_{1}\right)\cup f\left(\Gamma_{Q},A_{2}\right)$.

3. Let $\Psi_{Q}\left(b\right)\in f\left[\left(\Gamma_{Q},A_{1}\right)\cap \left(\Gamma_{Q},A_{2}\right)\right]$. Then $f\left(\Gamma_{Q}\left(a\right)\right)=\Psi_{Q}\left(b\right)$ for $\Gamma_{Q}\left(a\right)\in \left[\left(\Gamma_{Q},A_{1}\right)\cap \left(\Gamma_{Q},A_{2}\right)\right]$. By using intersection definition, we have $\Gamma_{Q}\left(a\right)\in \left(\Gamma_{Q},A_{1}\right)$ and $\Gamma_{Q}\left(a\right)\in \left(\Gamma_{Q},A_{2}\right)$. This implies $\Psi_{Q}\left(b\right)\in f\left(\Gamma_{Q},A_{1}\right)$ and $\Psi_{Q}\left(b\right)\in f\left(\Gamma_{Q},A_{2}\right)$. Hence, $\Psi_{Q}\left(b\right)\in f\left(\Gamma_{Q},A_{1}\right)\cap f\left(\Gamma_{Q},A_{2}\right)$. Thus, $f\left[\left(\Gamma_{Q},A_{1}\right)\cap \left(\Gamma_{Q},A_{2}\right)\right]\subseteq f\left(\Gamma_{Q},A_{1}\right)\cap f\left(\Gamma_{Q},A_{2}\right)$.

Conversely, suppose $\Psi_{Q}\left(b\right)\in f\left(\Gamma_{Q},A_{1}\right)\cap f\left(\Gamma_{Q},A_{2}\right)$. By using intersection definition, we have $\Psi_{Q}\left(b\right)\in f\left(\Gamma_{Q},A_{1}\right)$ and $\Psi_{Q}\left(b\right)\in f\left(\Gamma_{Q},A_{2}\right)$. This implies $\Psi_{Q}\left(b\right)=f\left(\Gamma_{Q}\left(a_{1}\right)\right)$ for some $\Gamma_{Q}\left(a_{1}\right)\in \left(\Gamma_{Q},A_{1}\right)$ and $\Psi_{Q}\left(b\right)=f\left(\Gamma_{Q}\left(a_{2}\right)\right)$ for some $\Gamma_{Q}\left(a_{2}\right)\in \left(\Gamma_{Q},A_{2}\right)$. Since $f\left(\Gamma_{Q}\left(a_{1}\right)\right)=f\left(\Gamma_{Q}\left(a_{2}\right)\right)=\Psi_{Q}\left(b\right)$, then $\Gamma_{Q}\left(a_{1}\right)=\Gamma_{Q}\left(a_{2}\right)$ if *f* is one to one. $\Psi_{Q}\left(b\right)\in f\left[\left(\Gamma_{Q},A_{1}\right)\cap \left(\Gamma_{Q},A_{2}\right)\right]$. Hence, $f\left(\Gamma_{Q},A_{1}\right)\cap f\left(\Gamma_{Q},A_{2}\right)\subseteq f\left[\left(\Gamma_{Q},A_{1}\right)\cap \left(\Gamma_{Q},A_{2}\right)\right]$. Thus, $f\left(\Gamma_{Q},A_{1}\right)\cap f\left(\Gamma_{Q},A_{2}\right)$= $f[\left(\Gamma_{Q},A_{1}\right)\cap \left(\Gamma_{Q},A_{2}\right)]$.

**Definition** **20.** Let $f:\left(\Gamma_{Q},A\right)\to \left(\Psi_{Q},B\right)$ and $g:\left(\Psi_{Q},B\right)\to \left(\Upsilon_{Q},C\right)$ be two Q-NS functions. Then $g\circ f:\left(\Gamma_{Q},A\right)\to \left(\Upsilon_{Q},C\right)$ is also a Q-NS function defined by $\left(g\circ f\right)\left(\Gamma_{Q}\left(a\right)\right)=g\left(f\left(\Gamma_{Q}\left(a\right)\right)\right)$.

**Definition** **21.** Let $f:\left(\Gamma_{Q},A\right)\to \left(\Psi_{Q},B\right)$ be a bijective function. Then the inverse relation $f^{-1}$: $\left(\Psi_{Q},B\right)\to \left(\Gamma_{Q},A\right)$ is called the inverse function.

Now we will define and propose a few theorems on the composition of Q-neutrosophic soft functions.

**Theorem** **6.** If $f:\left(\Gamma_{Q},A\right)\to \left(\Psi_{Q},B\right)$ is bijective then $f^{-1}:\left(\Psi_{Q},B\right)\to \left(\Gamma_{Q},A\right)$ is also a bijective function.

**Proof.**  Let $\Psi_{Q}\left(b_{1}\right)\neq \Psi_{Q}\left(b_{2}\right)$ for $\Psi_{Q}\left(b_{1}\right),\Psi_{Q}\left(b_{2}\right)\in \left(\Psi_{Q},B\right)$, and let $f^{-1}\left(\Psi_{Q}\left(b_{1}\right)\right)=\Gamma_{Q}\left(a_{1}\right)$ and $f^{-1}\left(\Psi_{Q}\left(b_{2}\right)\right)=\Gamma_{Q}\left(a_{2}\right)$. Then $f\left(\Gamma_{Q}\left(a_{1}\right)\right)=\Psi_{Q}\left(b_{1}\right)$ and $f\left(\Gamma_{Q}\left(a_{2}\right)\right)=\Psi_{Q}\left(b_{2}\right)$. Since *f* is one to one, $f\left(\Gamma_{Q}\left(a_{1}\right)\right)\neq f\left(\Gamma_{Q}\left(a_{1}\right)\right)$ implies $\Gamma_{Q}\left(a_{1}\right)\neq \Gamma_{Q}\left(a_{2}\right)$. Therefore, $f^{-1}\left(\Psi_{Q}\left(b_{1}\right)\right)\neq f^{-1}\left(\Psi_{Q}\left(b_{2}\right)\right)$. Hence $f^{-1}$ is one to one.

Now $\Gamma_{Q}\left(a\right)$ is an element of $\left(\Gamma_{Q},A\right)$. Since *f* is surjective, there exists a unique element $\Psi_{Q}\left(b\right)$ in $\left(\Psi_{Q},B\right)$ such that $f\left(\Gamma_{Q}\left(a\right)\right)=\Psi_{Q}\left(b\right)$ implies $\Gamma_{Q}\left(a\right)=f^{-1}\left(\Psi_{Q}\left(b\right)\right)$ for $\Gamma_{Q}\left(a\right)$ in $\left(\Gamma_{Q},A\right)$. Thus $f^{-1}$ is onto. Hence, $f^{-1}$ is bijective. ☐

**Theorem** **7.** Let $f:\left(\Gamma_{Q},A\right)\to \left(\Psi_{Q},B\right),g:\left(\Psi_{Q},B\right)\to \left(\Upsilon_{Q},C\right)$ be two bijective functions. Then $g\circ f:\left(\Gamma_{Q},A\right)\to \left(\Upsilon_{Q},C\right)$ is also a bijective and ${\left(g\circ f\right)}^{-1}=f^{-1}\circ g^{-1}$.

**Proof.**  Let $\Gamma_{Q}\left(a_{1}\right),\Gamma_{Q}\left(a_{2}\right)$ be two distinct elements of $\left(\Gamma_{Q},A\right)$. Since *f* and *g* are one to one we have $f\left(\Gamma_{Q}\left(a_{1}\right)\right)\neq f\left(\Gamma_{Q}\left(a_{2}\right)\right)$ and $g\left(f\left(\Gamma_{Q}\left(a_{1}\right)\right)\right)\neq g\left(f\left(\Gamma_{Q}\left(a_{2}\right)\right)\right)$. This implies $\left(g\circ f\right)\left(\Gamma_{Q}\left(a_{1}\right)\right)\neq \left(g\circ f\right)\left(\Gamma_{Q}\left(a_{2}\right)\right)$. Hence, g∘f is one to one.

Let $\Upsilon_{Q}\left(c\right)$ be an element of $\left(\Upsilon_{Q},C\right)$. Then there exists $\Psi_{Q}\left(b\right)$ in $\left(\Psi_{Q},B\right)$ such that $g\left(\Psi_{Q}\left(b\right)\right)=\Upsilon_{Q}\left(c\right)$ as *g* is onto. Again since *f* is onto there exists $\Gamma_{Q}\left(a\right)$ in $\left(\Gamma_{Q},A\right)$ such that $f\left(\Gamma_{Q}\left(a\right)\right)=\Psi_{Q}\left(b\right)$. Then, $\left(g\circ f\right)\left(\Gamma_{Q}\left(a\right)\right)=\Upsilon_{Q}\left(c\right)$ for every $\Upsilon_{Q}\left(c\right)$ in $\left(\Upsilon_{Q},C\right)$. Thus, g∘f is onto. Hence, g∘f is bijective. Since f,g and g∘f are bijective, they are invertible and for any relation $\left(R_{Q},C\right)$ and $\left(Z_{Q},C\right)$ we have ${\left(Z_{Q}\circ R_{Q}\right)}^{-1}=R_{Q}^{-1}\circ Z_{Q}^{-1}$, therefore ${\left(g\circ f\right)}^{-1}=f^{-1}\circ g^{-1}$. ☐

## 6. Decision Making Method

In this section, we present an application of Q-NSR in a decision-making problem.

The problem we consider is as below.

Let X={a,b,c,d} be a set of cars, Q={p,q} be the set of colors and *E* be a set of parameters where $E=\{e_{1}=cheap,e_{2}=type,e_{3}=modern,e_{4}=large\}$. If two individuals are going to buy a car according to their choice of parameters $A=\{e_{1},e_{4}\}$, $B=\{e_{2}\}$.

Suppose the Q-NSS $\left(\Gamma_{Q},A\right)$ describes the influence of being cheap and large on the degree of attraction of a car with a specific color and the Q-NSS $\left(\Psi_{Q},B\right)$ describes the influence of the type on the degree of attractivness of a car with a specific color as follows.

$$\begin{array}{cc} \left(\Gamma_{Q},A\right)=\{\langle e_{1}, & [(a,p),0.5,0.4,0.7],[(a,q),0.7,0.3,0.2],[(b,p),0.4,0.3,0.4],[(b,q),0.6,0.1,0.3],\\ & [(c,p),0.8,0.2,0.7],[(c,q),0.2,0.1,0.9],[(d,p),0.4,0.9,0.3],[(d,q),0.5,0.5,0.1]\rangle ,\\ \langle e_{4}, & [(a,p),0.9,0.7,0.3],[(a,q),0.1,0.4,0.9],[(b,p),0.2,0.1,0.1],[(b,q),0.5,0.3,0.4],\\ & [(c,p),0.3,0.2,0.2],[(c,q),0.4,0.7,0.7],[(d,p),0.6,0.3,0.5],[(d,q),0.3,0.9,0.1]\rangle \}. \end{array}$$

$$\begin{array}{cc} \left(\Psi_{Q},B\right)=\{\langle e_{2}, & [(a,p),0.7,0.2,0.6],[(a,q),0.2,0.3,0.5],[(b,p),0.9,0.6,0.5],[(b,q),0.3,0.2,0.1],\\ & [(c,p),0.5,0.7,0.9],[(c,q),0.5,0.7,0.6],[(d,p),0.9,0.8,0.7],[(d,q),0.8,0.6,0.4]\rangle \}. \end{array}$$

Then we can select a car on the basis of their parameters using Q-NSR, by applying the following algorithm.

**Step** **1.** Construct two Q-NSSs over *X*, $\left(\Gamma_{Q},A\right)$ and $\left(\Psi_{Q},B\right)$.
**Step** **2.** Construct a Q-NSR $\left(R_{Q},C\right)$ as requested.
**Step** **3.** Compute the comparison table using the formula: T+I−F.
**Step** **4.** Select the highest numerical grades for each column in the comparison table.
**Step** **5.** Find the score table which shows the objects with highest numerical grades corresponding to every pair of parameters.
**Step** **6.** Compute the score $S_{\left(x,q\right)}$ of each object (x,q) by taking the sum of those numerical grades.
**Step** **7.** The decision is any one of the elements in *M* where $M=\max_{(x,q)\in X\times Q}\left\{S_{\left(x,q\right)}\right\}$.

To execute the above steps, we will use the Q-NSSs $\left(\Gamma_{Q},A\right)$ and $\left(\Psi_{Q},B\right)$ to apply Step 2 to Step 7.

We obtain the Q-NSR $R_{Q}$ corresponding to the Cartesian product of $\left(\Gamma_{Q},A\right)$ and $(\Psi_{Q},B),$ respectively as follows.

$$\begin{array}{cc} R_{Q}=\{\langle \left(e_{1},e_{2}\right), & [(a,p),0.5,0.4,0.7],[(a,q),0.2,0.3,0.5],[(b,p),0.4,0.6,0.5],[(b,q),0.3,0.2,0.3],\\ & [(c,p),0.5,0.7,0.9],[(c,q),0.2,0.7,0.9],[(d,p),0.4,0.9,0.7],[(d,q),0.5,0.6,0.4]\rangle ,\\ \langle \left(e_{4},e_{2}\right), & [(a,p),0.7,0.7,0.6],[(a,q),0.1,0.4,0.9],[(b,p),0.2,0.6,0.5],[(b,q),0.3,0.3,0.4],\\ & [(c,p),0.3,0.7,0.9],[(c,q),0.4,0.7,0.7],[(d,p),0.6,0.8,0.7],[(d,q),0.3,0.9,0.4]\rangle \}. \end{array}$$

We compute the comparison table as:

The highest numerical grade for each column in Table 1 is written in bold, and the score is tabulated in Table 2.

The score of each object by taking the sum of these numerical grades are: $S_{\left(a,p\right)}=0.8$ and $S_{\left(d,q\right)}=0.8+0.7=1.5$.

Hence, $M=\max_{(x,q)\in X\times Q}\left\{S_{\left(x,q\right)}\right\}=S_{\left(d,q\right)}$. Thus, the decision is to choose the associated object (d,q) which represents car “*d*” of color “*q*”, as the appropriate solution for selecting the most suitable car according to the basis of parameters $e_{1},e_{4}$ and their related $e_{2}$. Therefore, the selection of a car is dependent on the choice parameters of each buyer.

## 7. Conclusions

This work established the notion of relations between Q-NSSs. It improved the concept of relations between fuzzy sets, soft sets and hybrid models of these two types of sets because the relations have been constructed based on the Q-NSSs model which has three independent two-dimensional membership functions for uncertainty, indeterminacy and falsity. It provided an adequate parametrization tool to deal with the aspects of two-dimensional imprecise, indeterminate and inconsistent data which appear in most real life problems. The notions of inverse, composition of Q-NSRs and functions were also presented along with some related theorems and properties. The ideas of reflexive, symmetric, transitive and equivalence Q-NSRs were defined followed by the introduction of the associated properties with illustrative examples. An algorithm using Q-NSR was constructed and applied to a decision making problem. Q-NSS facilitates the way to numerous prospects for future research since it serves the two-dimensionality and indeterminacy simultaneously. Thus, it can be extended using the refined neutrosophic set [40], soft expert set [11] and many other structures. The structure of the Q-NSR enables it to describe the relations between two-dimensional imprecise, indeterminate and inconsistent data. It may be applied to different branches of mathematics, especially to graph theory, to establish the notion of Q-neutrosophic graphs. It also may provide a powerful framework to represent problems with uncertainty and two-dimensionality simultaneously in the fields of physics, engineering and medical diagnosis.

## Figures and Tables

**Table 1:** Comparison table.

*X* × *Q*	$\left(e_{1},e_{2}\right)$	$\left(e_{4},e_{2}\right)$
(a,p)	0.2	**0.8**
(a,q)	0	−0.4
(b,p)	0.5	0.3
(b,q)	0.2	0.2
(c,p)	0.3	0.1
(c,q)	0	0.4
(d,p)	0.6	0.7
(d,q)	**0.7**	**0.8**

**Table 2:** Score table.

$\left(e_{1},e_{2}\right)$	$\left(e_{4},e_{2}\right)$
(d,q)	(a,p),(d,q)
0.7	0.8
